# Annotations

**Published:** 1889-01-19

**Authors:** 


### Annotations.

**Desperate Diseases—Desperate Remedies.**

In medical practice there are well-known methods of treatment which are very severe, but which are held to be justified by results. For example, one patient will rather endure the shock of tooth-extraction than the agony of tooth-ache; another will submit to the torture of a mustard-plaster for half-an-hour in order to counteract the ill effects of a deep-seated inflammation; a third will lie down quietly on the operation-table, and permit the surgeon to amputate a leg or an arm in order that he may live a little longer, though in a mutilated and disabled condition. Why, then, in the name of plain reason, are we so exceedingly tender to the diseases and ills of the people in the mass, when we are so relentlessly severe with ourselves as individuals? It will be granted that burglary is a social disease—that an armed burglar is a disease "germ" of the most immediately dangerous kind. Yet where in the Statute-book are our tooth-extracting instruments, our mustard-blisters, our surgeon's knives, and saws, and tourniquets, for the counter-irritation or amputation of those parts of the body politic which are represented by armed and unarmed burglars? Our point is this, that as individuals we can be as severe to ourselves as the circumstances need, and that we make no fuss about it; but as a people we do not adapt means to ends in the same scientific and experimental way. What, for instance, should be done to the three armed and desperate ruffians who attacked Mr. Atkin's house at Muswell Hill last week, who shot one man nearly to death, and were quite prepared to shoot a dozen or a score if it had been necessary? There is only one proper kind of treatment for such men which will be at all deterrent, and that is flogging. Let such scoundrels be sentenced to ten years' penal servitude, and let each of them have twenty, thirty, or fifty lashes every six months during their term of imprisonment. By the time they have had their second or third dose they will have begun to understand what it is not only to have their hand against every man, but every man's hand against them. They will know what daily dread and nightly terror are. They will understand the tortures which an excited imagination can inflict. Once let a few of them be treated thus, and the fraternity will soon learn rather to endure a lifetime of penal servitude than to attempt to fire a single shot; rather to risk death by jumping into the nearest river than to risk such tortures for the sake of freedom to rob and murder. Desperate diseases insist upon desperate remedies, and only in such remedies is there any hope of cure.

**Impossible!**

A morning contemporary reports certain facts concerning the recent analysis of a sample of milk by public officials which ought to make the very door-plates of medical analysts blush for shame, if, as Hood has it, "door-plates were not so brazen." A dairyman was charged at the Wandsworth Police Court with selling adulterated milk. His conviction or acquittal depended, of course, upon the evidence of scientific experts. Was the milk adulterated? that was the question. Yes, said Dr. Cassal, the public analyst; it was adulterated to the extent of 9 per cent. Not quite so bad as that, said the Somerset House people; it was only adulterated to the extent of 4 per cent. No, said Dr. Attwell, it was not adulterated at all. The magistrate, considering the great discrepancy of the evidence, dismissed the summons. It is clear from these facts that the analysis of milk, at any rate, has not reached any very high standard of scientific accuracy. None of us are surprised at that, however much disappointed we may be. The usual tests for milk are known to be of a very rough-and-ready character. The question, however, is this: Is it not possible to devise methods of testing the composition of milk which shall be at once simple, accurate, and convincing? And if it is not, then is it proper that analysts of no more than ordinary skill should have the power to secure a milkman's conviction, to destroy his character as an honest man, and to ruin his business? It is not proper: it is a bad statute which permits of such consequences, and it ought to be repealed or amended. But a larger question is raised than that of milk adulteration. It is the question of the reliability and the degree of accuracy of chemical analysis in general. Chemistry is a wonderful science. In it the operations of analysis and synthesis may be carried out with a measure of accuracy which is truly admirable and amply sufficient for many practical purposes. But how many chemical processes can be relied upon as mathematically correct? That is a crucial point, and it is not seldom a point of the utmost possible practical importance. The general public have the idea that a chemical problem may be solved as easily and as accurately as a mathematical question. On paper it may, but not always in the laboratory. It may be solved as easily, but never with such absolutely reliable precision. All men love to be infallible, and to speak with authority, chemists as well as others. But the great world ought to know that in every chemical laboratory mistakes may and do occur; and that in many analytical processes the only conclusions that can be arrived at are such as are, relatively and generally, not absolutely and precisely correct. The chemist, like the doctor, does his best. His best is often wonderfully correct and complete; but chemical science is not mathematical science, and probably never can be, to men and women. No doubt in itself every fact in every science is as precise and accurate as are mathematical processes; but human beings have not got the necessary mental apparatus for analysing and synthetising without possibility of mistake. These limitations should never be forgotten in everyday affairs.

**A Sheffield "Scare."**

It is not a thing to be wondered at that uninstructed people should "fight shy" of hospitals for infectious diseases. In olden times the same class had a lively dread of ghosts. It must be admitted that the terror of infectious diseases has a more rational foundation than the fear of the wandering spirits of the midnight hour. But every one will admit that "fear" is an unplesant companion, and that baseless fear, in addition to being unpleasant, is also ridiculous. The Sheffield people have had more than one "scare" about their Borough Hospital for Infectious Diseases, situated in Winter Street, and now the feeling has been once more revived by active spirits bent upon movement, if not upon mischief. For ourselves we entirely concur in the determination arrived at by some, that if they can help it no one part of Sheffield shall be subjected to greater risks from infectious diseases than any other part. It is not reasonable that one district shall have a concentration of dangers forced upon it in order that every other district may be the more free. With the spirit that may be presumed to animate the "discontents" we have no controversy. But zeal should always be tempered by knowledge; and the greater the zeal the more abundant and accurate should be the knowledge. It is here where the Sheffield objectors are so conspicuously lacking. Mr. Percy Rawson gives ample proof of zeal, whilst evidence as to knowledge on his part is entirely *nil*. It happily does not require either a long or a learned argument to show the Sheffield people who live in the neighbourhood of Winter Street that their fears as to the danger of infection from their Borough Hospital may for the future be considered quite groundless. The managers have decided that small-pox cases shall not be treated there; and with the disappearance of small-pox cases danger vanishes. It is difficult to imagine any proofs more convincing than those that have been adduced in support of the harmlessness of an ordinary fever hospital, when small-pox cases are excluded. All instructed medical opinion is on one side of the question, and that side proclaims the absolute freedom from danger to those who live outside the hospital walls. A Royal Commission has taken abundant evidence, and medical officers of health have concurred in the expression of the most decided opinions. In this case doctors do not disagree; they are all of one mind. Not only so, but the conclusions arrived at have been obtained by methods which appeal to every man's intelligence. They are the result of experience, of experience gathered by trained observers and adjudicated upon by trained judges. Mere hypothesis is altogether excluded. The thirty years' history of different infectious hospitals situated in crowded districts of London and provincial towns has been carefully searched and collated, and the evidence of living officials has been taken. There is an absolute consensus of opinion and statement that when small-pox cases are excluded, fever hospitals add no risks whatever to any neighbourhood in which they may be placed. The Sheffield people may "take comfort," and "leave well alone."

